# An Indoor Fingerprint Positioning Algorithm Based on WKNN and Improved XGBoost

**DOI:** 10.3390/s23083952

**Published:** 2023-04-13

**Authors:** Haizhao Lu, Lieping Zhang, Hongyuan Chen, Shenglan Zhang, Shoufeng Wang, Huihao Peng, Jianchu Zou

**Affiliations:** 1 College of Mechanical and Control Engineering, Guilin University of Technology, Guilin 541006, China; lo726997100@126.com (H.L.); 1994023@glut.edu.cn (L.Z.);; 2 Department of Electrical and Electronic Engineering, Guilin University of Technology AT Nanning, Nanning 532100, China; 3 Key Laboratory of AI and Information Processing, Education Department of Guangxi Zhuang Autonomous Region, Hechi University, Yizhou 546300, China

**Keywords:** WKNN, indoor localization, WiFi fingerprint, XGBoost, genetic algorithm

## Abstract

Considering the low indoor positioning accuracy and poor positioning stability of traditional machine-learning algorithms, an indoor-fingerprint-positioning algorithm based on weighted k-nearest neighbors (WKNN) and extreme gradient boosting (XGBoost) was proposed in this study. Firstly, the outliers in the dataset of established fingerprints were removed by Gaussian filtering to enhance the data reliability. Secondly, the sample set was divided into a training set and a test set, followed by modeling using the XGBoost algorithm with the received signal strength data at each access point (AP) in the training set as the feature, and the coordinates as the label. Meanwhile, such parameters as the learning rate in the XGBoost algorithm were dynamically adjusted via the genetic algorithm (GA), and the optimal value was searched based on a fitness function. Then, the nearest neighbor set searched by the WKNN algorithm was introduced into the XGBoost model, and the final predicted coordinates were acquired after weighted fusion. As indicated in the experimental results, the average positioning error of the proposed algorithm is 1.22 m, which is 20.26–45.58% lower than that of traditional indoor positioning algorithms. In addition, the cumulative distribution function (CDF) curve can converge faster, reflecting better positioning performance.

## 1. Introduction

As urban modernization has been continuously improved, the demand for spatial coordinate information has been constantly increasing, during which the development of positioning technologies has attracted extensive attention from numerous researchers. In complex indoor environments such as supermarkets, subway stations, and underground garages with complicated spatial structures, it is so difficult to receive accurate satellite signals that satellite positioning systems such as global positioning system (GPS) fail to meet the needs for accurate positioning in indoor environments [1]. The current indoor positioning signals commonly used are radio frequency identification (RFID), ultrasonic wave, ultra-wide band (UWB), WiFi, etc., among which WiFi signals are widely used due to the advantages of long transmission distance, low cost, high fidelity, relatively perfect WiFi arrangement in most environments, and easy acquisition.

Given the spatial difference of wireless signals (or other environmental characteristics) in different environments, WiFi-based fingerprint positioning is used to establish a position–fingerprint relationship database with the wireless signal feature at a specific spatial position as the fingerprint of this position, thus estimating the user’s position by means of fingerprint matching [2]. In the offline database building stage, the multi-dimensional vectors of RSSI values are collected from various wireless access points (APs) in a certain range and connected to the identified coordinate positions, and a fingerprint database is built using such measurement results. In the online position matching stage, the RSSI of WiFi collected by the user device is matched with the information of the fingerprint database using an intelligent algorithm to acquire the user’s position information at the time so as to realize positioning. The WiFi-based indoor-fingerprint-positioning accuracy is easily affected by the amount of fingerprint information in the fingerprint database, the actual environmental interference, and the positioning algorithm. Relevant studies have been extensively carried out in China and abroad to further improve the accuracy and stability of WiFi-based indoor fingerprint positioning. Aiming at the large calculated quantity of the k-nearest neighbor (KNN) algorithm, Wu Z. et al. [3] fused the KNN and clustering ideas for combined optimization by improving the similarity calculation method. Oluwaseyi Paul Babalola [4] put forward a feature extraction method based on dynamic mode decomposition for the hidden Markov model to reduce indoor environmental interference and improve positioning accuracy. Xiang W. et al. [5] proposed a KNN positioning algorithm based on entropy weights, which improved the positioning accuracy by adaptively adjusting the weight ratio of each matched sampling point with the entropy weight method. Rohan Kumar Yadav [6] introduced the credible KNN Bayesian estimation of the system and integrated a low-power Bluetooth beacon to improve positioning accuracy. Though effectively improving the indoor positioning accuracy, the improved KNN algorithm fails to solve the low anti-noise ability and poor stability in the positioning process. Hence, efforts have been made by some scholars to optimize fingerprint signal processing. WAFA NJIMA [7] generated false fingerprints based on generative adversarial networks, which considerably reduced the time consumption and workload of fingerprint collection and improved the positioning accuracy by 10.11%. Pan W. et al. [8] performed permutation and combination using the linear discrimination algorithm in a low-dimensional case, summed the obtained probability values, and set constraints through thresholds to improve the accuracy of adjacent grid positioning. Bundak C. et al. [9] optimized a database through the clustering method, matched the top 10 reference points (RPs) of the nearest Euclidean distance with the target points (TPs) with RP clustering, and found the final estimated position through the average Euclidean algorithm. Zhou C. et al. [10] put forward an unscented particle filter-aided fusion positioning method based on WiFi and PDR positioning to perform robust adaptive optimization of the particle filter and further improve the positioning accuracy. Ninh D B [11] came up with a random statistical method to process the noise of WiFi signals in the offline stage and standardize the position fingerprint database, and determined the indoor position using the Mahalanobis distance during online positioning, thereby enhancing the positioning accuracy of the random statistical method. The weighted KNN (WKNN) algorithm can effectively improve the classification performance of the KNN algorithm by assigning different weights to the K nearest neighbors of the test sample according to the different distances between the two, where the maximum weight is assigned to the nearest neighbor closest to the test sample. However, the WKNN algorithm has the disadvantages of large time consumption and low accuracy when processing the RSSI difference [12]. Considering the inaccurate weighting of RPs by the traditional WKNN algorithm using the reciprocal of the RSSI difference, Pheng S [13] proposed an improved WKNN algorithm based on the physical distance weighting of RSSI. Bundak C E A [14] put forward an improved WKNN algorithm which, without a fixed K value, established a KNN model via support vector regression to determine the K value of each specific position, achieving higher positioning accuracy.

Machine-learning algorithms can predict the coordinate values of target locations in indoor fingerprint localization using only information such as signal strength. Many machine-learning techniques have been used to improve localization performance [15]. However, most implementations of indoor localization prediction currently rely on a single machine-learning algorithm. Moreover, indoor positioning is easily affected by noise signals and the mutual interference between them, which leads to the reduction of positioning accuracy [16]. Considering the above issues, an indoor fingerprint localization algorithm based on WKNN and XGBoost is proposed in this study by integrating their advantages in indoor localization. The following contributions were mainly drawn in this study:

1. In view of the shortcomings of the traditional KNN positioning algorithm in practical application, the WKNN algorithm was fused with the XGBoost algorithm in this study to form a positioning algorithm based on WKNN and XGBoost, which improved the large ranging error induced by external interference.
2. The influences of such parameters as the K value, the number of regression trees, the depth of decision trees, and the learning rate in the XGBoost algorithm were investigated, and such parameters as the number of regression trees, the depth of decision trees, and the learning rate in the XGBoost algorithm were optimized through the genetic algorithm (GA).
3. The corresponding verification experiment was designed. Then, the proposed algorithm was compared with the KNN, WKNN, XGBoost, and WKNN+XGBoost algorithms in the other literature regarding the positioning effect, which was evaluated using the positioning error, root-mean-square error (RMSE), mean absolute error (MAE), and cumulative distribution function (CDF) of probability. Finally, the superiority of the proposed algorithm was verified.

The remainder of this study was organized as follows: In Section 2, the KNN, WKNN, and XGBoost algorithms were introduced, and the influences of important parameters on such algorithms were analyzed. In Section 3, the flow of GA and fusion algorithm was expounded. In Section 4, experimental verification was conducted and the experimental results were analyzed. In Section 5, conclusions were drawn and expectations were presented.

## 2. Introduction of Relevant Basic Algorithms

### 2.1. KNN and WKNN Algorithms

We assumed that L fingerprints were arranged in the offline stage of fingerprint positioning, which were denoted as $\{F_{1},F_{2},\ldots ,F_{l}\}$, with their corresponding coordinates expressed as $\{L_{1},L_{2},\ldots ,L_{l}\}$. After the fingerprint database was established, the fingerprint at the undetermined position was recorded as *S*, which was the average value of RSS obtained by all APs after repeated measurements, that is, $S=\{s_{1},s_{2},\ldots ,s_{n}\}$. All fingerprints were stored in the form of $F_{i}=\{r_{1}^{i},r_{2}^{i},\ldots ,r_{l}^{i}\}$, which stands for the value taken for the 1-*n*-th AP in the *i*-th fingerprint. By adopting Euclidean distance as shown in Formula (1), the distance difference between the coordinates of the fingerprint *S* at the undetermined position and the data collected in the offline fingerprint database could be obtained:

(1) $$w_{1}=\sqrt{\overset{n}{\underset{i=1}{\sum }}{\left(s_{i}-r_{i}\;\right)}^{2}}$$

where n, $s_{i}$, and $r_{i}$ represent the n-th AP, the predicted value, and the actual value, respectively.

Then, the minimum value was taken as the estimated position according to the distance differences, which was averaged to obtain the final result, as seen in Formula (2):

(2) $$L=\left(\hat{x},\hat{y}\right)=\frac{1}{K}\overset{k}{\underset{i=1}{\sum }}\left(x_{i},y_{i}\right)$$

where K denotes K nearest neighbor points, and $\left(x_{i},y_{i}\right)$ stands for the coordinates of each nearest neighbor point.

The signal strength of WiFi decreases gradually with the increase in distance, so a greater distance between the TP to be measured and the RP leads to a stronger signal fluctuation of the RP and a smaller positioning effect. However, the traditional KNN assigns the same weight to all nearest neighbors and tends to ignore the fingerprint information at a long distance, which affects the indoor positioning accuracy.

Different from the traditional KNN algorithm that assigns the same weight to all nearest neighbors, the WKNN algorithm enhances the local contribution ability between nearest neighbors by giving different weights to samples based on the distance, which can further improve the accuracy [17]. In this study, therefore, the WKNN algorithm was adopted as the fingerprint positioning algorithm. With similar steps to the KNN algorithm, the WKNN algorithm adds the distance-based weight on the basis of Formula (3).

In the case of WKNN algorithm-based positioning, the coordinates of the TP can be expressed as Formula (3):

(3) $$L_{1}=\left(\hat{x},\hat{y}\right)=\frac{1}{\overset{k}{\underset{i=1}{\sum }}\left(w_{i}+\varepsilon \right)}\overset{k}{\underset{i=1}{\sum }}\left(x_{i}(w_{i}+\varepsilon ),y_{i}(w_{i}+\varepsilon )\right)$$

where *K* represents the number of nearest neighbor RPs, and *ε* is a small non-zero real number, aiming to avoid $w_{i}$ = 0. The weight formula is shown in Formula (4):

(4) ωi=1R∑i=1n1R

where *R* stands for the distance from the target, and a larger value indicates a smaller influence and weight. On the contrary, a smaller value means a greater influence and weight.

### 2.2. XGBoost Algorithm

XGBoost develops from gradient boosting decision tree (GBDT) [18], which abides by the main idea of integrating and outputting the prediction results of multiple trees, specifically as follows:

For a sample fingerprint dataset, the XGBoost algorithm was used to predict coordinates, thus obtaining the predicted coordinates $\overset{\wedge }{p_{i}}=(\overset{\wedge }{x_{i},}\overset{\wedge }{y_{i}})$. *k* decision trees were assumed in an XGBoost model, and the prediction result of this model on sample *i* is shown in Formula (5):

(5) $${\hat{y}}_{i}{}^{\left(k\right)}=\overset{K}{\underset{k=1}{\sum }}f_{k}\left(x_{i}\right)$$

where $f_{k}\left(x_{k}\right)$ denotes the regression value on a certain tree, $x_{i}$ stands for the eigenvector corresponding to sample *i*, and $f_{k}$ represents the *k*-th decision tree.

The objective function is generally expressed in a form of Formula (6), which includes two main parts, regularization and training loss:

(6) $$\Phi =\overset{n}{\underset{i=1}{\sum }}l\left(y_{i},{\hat{y}}_{i}\right)+\gamma T+\frac{1}{2}\lambda \overset{T}{\underset{J=1}{\sum }}\omega_{j}^{2}$$

where $\overset{n}{\underset{i=1}{\sum }}l\left(y_{i},{\hat{y}}_{i}\right)$ is the loss function and *n* represents the number of samples. In the second half of the formula, $\gamma T+\frac{1}{2}\lambda \overset{T}{\underset{J=1}{\sum }}\omega_{j}^{2}$ is the regularization part of the XGBoost model, which decides the depth of the tree. Therein, *γ* is a regularization parameter, i.e., the penalty term removed in the case of the increase in the number of leaves, and $\frac{1}{2}\lambda \overset{T}{\underset{J=1}{\sum }}\omega_{j}^{2}$ represents the least square error of the weight of each leaf node, in which *λ* is a regularization parameter, *T* stands for the number of leaf nodes, and $\omega_{j}$ represents the leaf weight of the *j*-th leaf node.

After parameter substitution, the objective function of the model is shown in Formula (7), where ${\hat{y}}_{i}^{\left(k-1\right)}$ is the iterative result of all trees. Therefore, ${\hat{y}}_{i}^{\left(k\right)}$ can be equivalent to ${\hat{y}}_{i}^{\left(k-1\right)}+f_{k}\left(x_{i}\right)$ upon the *k*-th iteration:

(7) $$\phi_{\left(k\right)}=\overset{n}{\underset{i=1}{\sum }}l\left(y_{i},{\hat{y}}_{i}^{\left(k-1\right)}+f_{k}\left(x_{i}\right)\right)+\gamma T+\frac{1}{2}\lambda \overset{T}{\underset{J=1}{\sum }}\omega_{j}^{2}$$

where $f_{k}\left(x_{i}\right)$ denotes the *k*-th iteration result of the tree.

After Taylor’s expansion, the objective function could be simplified to the form as shown in Formula (8):

(8) $$\phi_{\left(k\right)}{}^{*}=-\frac{1}{2}\overset{T}{\underset{j=1}{\sum }}\frac{G_{j}}{H_{j}+\lambda }+\gamma T$$

where $G_{j}=\underset{i=I_{j}}{\sum }g_{i}$ and $H_{j}=\underset{i=I_{j}}{\sum }h_{i}$, in which $I_{j}$ stands for each sample set of the j-th tree.

In the tree-building process of the XGBoost algorithm, all split sub-nodes in the tree model were traversed through the greedy algorithm, and the leaf node with the maximum gain of the objective function after splitting was chosen for splitting once again.

$I_{L}$ and $I_{R}$ were set to be the datasets contained by the sub-branch points at two sides of the branch after branching, so the objective function before splitting is shown in Formula (9):

(9) $$Obj_{1}=-\frac{1}{2}\frac{{\left(G_{L}+G_{R}\right)}^{2}}{H_{L}+H_{R}+\lambda }+\gamma$$

The split objective function is shown in Formula (10):

(10) $$Obj_{2}=-\frac{1}{2}\left[\frac{G_{L}{}^{2}}{H_{L}+\lambda }+\frac{G_{R}{}^{2}}{H_{R}+\lambda }\right]+\;2\gamma$$

For the objective function, therefore, the gain after splitting is displayed in Equation (11):

(11) $$\mathrm{Gain}=\frac{1}{2}\left[\frac{G_{L}{}^{2}}{H_{L}+\lambda }+\frac{G_{R}{}^{2}}{H_{R}+\lambda }-\frac{{\left(G_{L}+G_{R}\right)}^{2}}{H_{L}+H_{R}+\lambda }\right]-\gamma$$

From the above analysis, it could be seen that the final prediction result would be substantially influenced by the number, depth, and learning rate of trees in the XGBoost algorithm.

## 3. Design of Indoor-Fingerprint-Positioning Algorithm Based on WKNN and GA-Optimized XGBoost

### 3.1. GA

As a global heuristic optimization algorithm, GA has exhibited good effects in solving nonlinear problems such as parameter optimization, machine learning, signal processing, and automatic control, and been favored by many researchers so far [19]. The basic steps of GA are as below:

Step 1: The initial population is randomly generated. 

Step 2: Parameters are encoded through an appropriate encoding method. 

Step 3: The individual fitness value is set, and the probability for an individual to be chosen is calculated as per Formula (12):

(12) $$p(x_{i})=\frac{f(x_{i})}{\sum_{j=1}^{n}f(x_{j})}$$

where $p(x_{i})$ indicates the probability for each individual to be selected, and $f(x_{i})$ denotes the set individual fitness value.

Step 4: The individual cumulative fitness value $F(x_{i})$ and the individual cumulative selection probability $P(x_{i})$ are calculated according to Formula (13). Then, the crossover probability and mutation probability are solved through Formulas (14) and (15):

(13) $$\left\{ \begin{array}{l} F(x_{i})=\sum_{j=1}^{i}f(x_{j});\\ P(x_{i})=\sum_{j=1}^{i}p(x_{j}); \end{array}\right.$$

(14) $$p_{c}=\left\{ \begin{array}{l} p_{c}\frac{\left(f_{\max}-f^{\prime }\right)}{f_{\max}-f_{avg}},(f^{\prime }\geq f_{avg})\\ p_{c},(f^{\prime }\leq f_{avg}) \end{array}\right.$$

(15) $$p_{m}=\left\{ \begin{array}{l} p_{m}\frac{\left(f_{\max}-f^{\prime \prime }\right)}{f_{\max}-f_{avg}},(f^{\prime \prime }\geq f_{avg})\\ p_{m},(f^{\prime \prime }\leq f_{avg}) \end{array}\right.$$

where $f_{\max}$ is the fitness of the best individual in the population, $f_{avg}$ denotes the average fitness of the population, $f^{\prime }$ represents the maximum fitness of the two individuals to be crossed over, and $f^{\prime \prime }$ is the fitness of the individuals to be mutated.

GA algorithm is relatively immune to the local extremum in comparison with other optimization algorithms. With such an advantage, GA can be combined with machine-learning algorithms so that its advantage can be exerted to the fullest. In this study, therefore, more proper parameters such as the number, depth, and learning rate of trees were chosen for the XGBoost algorithm by taking the advantage of GA in global optimization.

### 3.2. Implementation of Indoor-Fingerprint-Positioning Algorithm Based on WKNN and GA-Optimized XGBoost

In the case of a large difference in sample size, the WKNN algorithm only calculates the neighbor nodes of the class containing a large number of samples while ignoring the node information with a small sample size, which will lead to data losses. However, the XGBoost algorithm, which integrates the merits of simple calculation and strong interpretability, is relatively suitable for samples with missing attribute values and applicable to complex and changeable indoor environments. Therefore, the WKNN and XGBoost algorithms were fused in this study following the idea of fusion models, and an indoor-fingerprint-positioning algorithm based on WKNN and improved XGBoost was proposed. For this proposed algorithm, such parameters as the learning rate of the XGBoost were optimized through GA, and then WKNN was integrated into the XGBoost model to obtain the final coordinates. The specific implementation process of the proposed algorithm was as follows:

Step 1: In the offline stage, the indoor fingerprint information was collected and the corresponding database was established. In this study, a total of n fingerprint collection points were arranged in the indoor experimental scenario. Therein, a piece of fingerprint data could be established when the *i*-th RP collected, for the *j*-th times, the signal strength sent by the AP, and its AP feature could be denoted in the form of Formula (16):

(16) $$r^{ij}=\left(RSSI_{i1},RSSI_{i2},\ldots ,RSSI_{ij}\right)$$

where $r^{ij}$ represents the RSSI value collected by the *i*-th RP totally for *j* times.

The original fingerprint database composed of the RSSI signals of n fingerprint RPs is shown in Formula (17):

(17) $$S=\left(r^{1},r^{2},\ldots ,r^{n}\right)$$

where $r^{i}=\left(r^{i1},r^{i2},\ldots r^{ij}\right)$, which indicates all fingerprint data collected by the *i*-th RP.

Step 2: The data in the offline fingerprint database were normalized and filtered through the Gaussian filtering algorithm to further reduce the data error of the collected offline fingerprint database.

Step 3: The weight was set according to the distance in the sample set, that is, a greater distance from the target represented its smaller influence and weight. On the contrary, a smaller distance from the target indicated its greater influence and weight. The weight definition method is shown in Formula (4) of Section 2.1.

Step 4: With XGBoost as the basic model, modeling was performed using the data of each $AP_{i}$, and an initially estimated coordinate value could be obtained based on the established model, as seen in Formula (18). Specific to the estimated value, the predicted coordinate value $\overset{\wedge }{p_{i}}=(\overset{\wedge }{x_{i},}\overset{\wedge }{y_{i}})$ on the test sample was calculated using the XGBoost model:

(18) $$C_{i}=f_{i}\left(AP_{ij},x,y\right)$$

Step 5: The population was initialized, its fitness function was determined, and its fitness value was calculated, as seen in Formula (19):

(19) $$f=\sqrt{\frac{1}{n}\overset{n}{\underset{i=1}{\sum }}{\left({\hat{x}}_{i}-x_{i}\right)}^{2}+{\left({\hat{y}}_{i}-y_{i}\right)}^{2}}$$

where *f* represents the fitness of the population.

Step 6: According to the fitness value, the individuals with higher fitness were selected for the subsequent crossover and mutation operations, and then the results of the new generation of population were output. The parameters of the XGBoost model were optimized through GA, so as to obtain the optimal parameter combination.

Step 7: Whether the population met the end condition was judged. If yes, the model results, i.e., the number of regression trees, the depth of decision trees, and the learning rate in the XGBoost algorithm, were output. Otherwise, the implementation should be started again from Step 6.

Step 8: Each sample in the test sample set needed training and tests in the WKNN algorithm model to find the nearest neighbor set of each sample and perform prediction in it. Then, the X and Y coordinate values and training errors of the model set on WKNN could be calculated, respectively. The parameters obtained in Step 7 were substituted into the XGBoost algorithm for prediction, and the coordinate values and training errors were calculated.

Step 9: The X and Y coordinate values and training errors of the model set on WKNN and improved XGBoost were output after the end conditions were met.

Step 10: The prediction model set was set as $P=\{P_{1},P_{2},\ldots ,P_{m}\}$. For each coordinate to be predicted, the training errors in the nearest neighbor set were predicted and identified through the model. Next, the weight was assigned to each prediction model on the basis of training errors, as seen in Formula (20):

(20) $$w_{i}=1/RMSE_{i}$$

where *RMSE* was calculated based on the selected nearest neighbor dataset.

**Figure 1:**
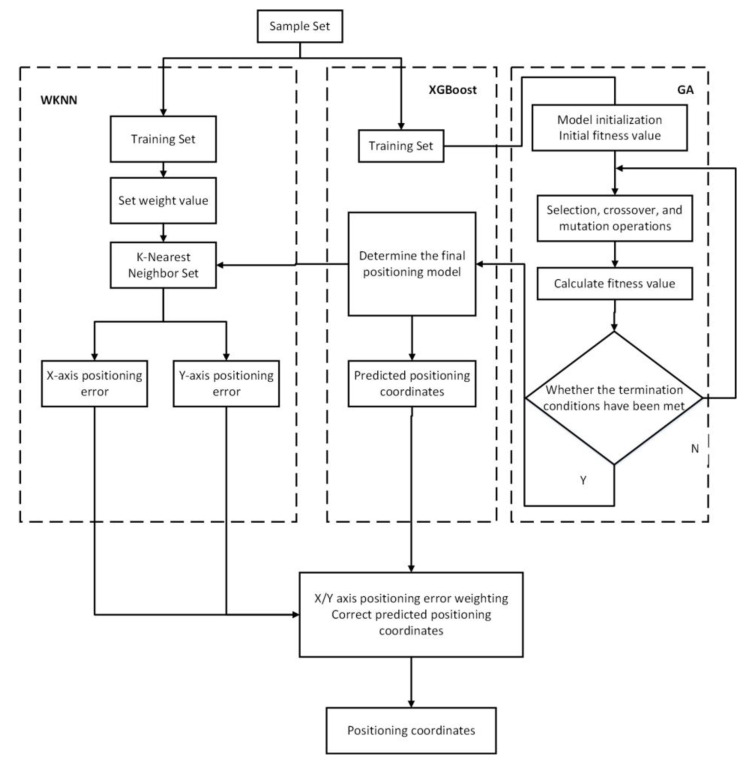
Indoor fingerprint positioning algorithm based on WKNN and GA-optimized XGBoost.

Step 11: According to the weight value obtained in Step 10, the prediction results in WKNN and the improved XGBoost fusion algorithm were combined and finally returned to the predicted coordinate value. The entire process diagram is shown in Figure 1.

## 4. Experiments and Result Analysis

### 4.1. Acquisition of WiFi Fingerprint Information

**Figure 2:**
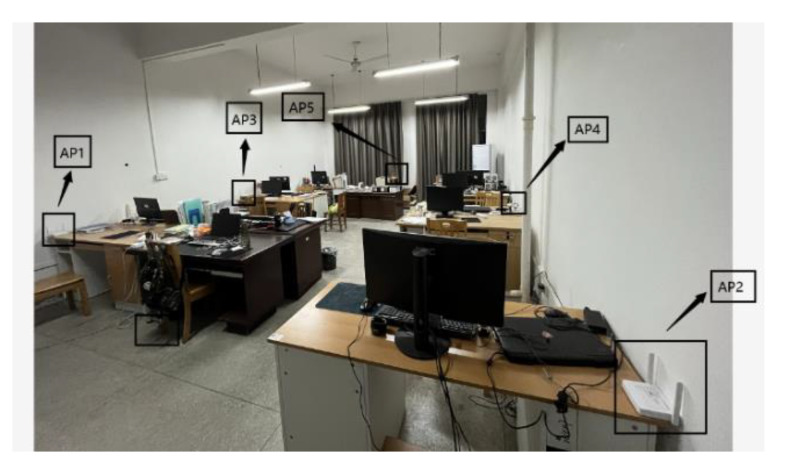
Realistic view of the experimental environment.

In the experiment, a MERCURY-MW305R router was used as the WiFi signal transmitting terminal, and a mobile phone with independently developed RSSI information acquisition software as the receiver, in the computer environment of Intel’s i5-6300HQ processor and Microsoft’s Windows 10 operating system (Professional Edition) in the United States. The studio used in the simulation experiment covered an area of about 5 m × 10 m. In the positioning area, a total of 5 APs about 0.8 m off the ground were deployed. The laboratory environment and AP positions are shown in Figure 2. The experimental area, in which there were 100 RPs, was gridded. The RSSI values of five WiFi hotspots were collected at each node, processed through Gaussian filtering, and averaged to build a fingerprint database.

After the deployment of RPs, signal acquisition and storage were implemented in the studio using the mobile device with independently developed RSSI information acquisition software as the receiver, which were finished after the whole RP set was traversed. Then, the collected fingerprint information was stored in the fingerprint database in the form of vectors, and then the fingerprint database (Table 1) was established based on the fingerprint information and the corresponding n-dimensional coordinates in the physical space. It could be seen that the fingerprint database mainly included the RSSI value of each AP collected from each RP and the recorded physical coordinate information of RPs.

### 4.2. Data Preprocessing of the Fingerprint Database

When influenced by some external factors, WiFi signals in different periods will fluctuate, which will lead to the unstable RSSI value of WiFi signals and interfere with the subsequent positioning. Before online use, therefore, the fingerprint database should be preprocessed to reduce the data error, enhance the data stability, and improve the online positioning accuracy. In this experiment, the Gaussian filtering algorithm was used as the filtering algorithm.

**Figure 3:**
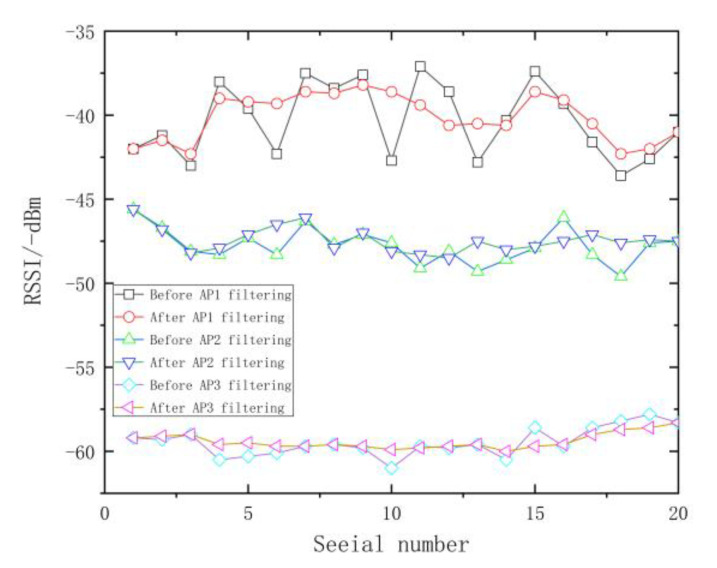
Comparison of fingerprint signal before and after filtering.

The 20 groups of RSSI data collected by AP1, AP2, and AP3 at a fixed RP were processed by Gaussian filtering on the MATLAB platform. The experimental results are shown in Figure 3, in which the horizontal axis denotes the number of acquisition times and the vertical axis denotes the signal strength value acquired. It could be observed from Figure 3 that after filtering, the RSSI data interval was evidently reduced, and its fluctuation was obviously reduced too. After Gaussian filtering, the interference data with a small probability in the fingerprint database could be removed, thus effectively inhibiting the influence of interference data on the experimental results.

### 4.3. Research on Algorithm Parameters

**Figure 4:**
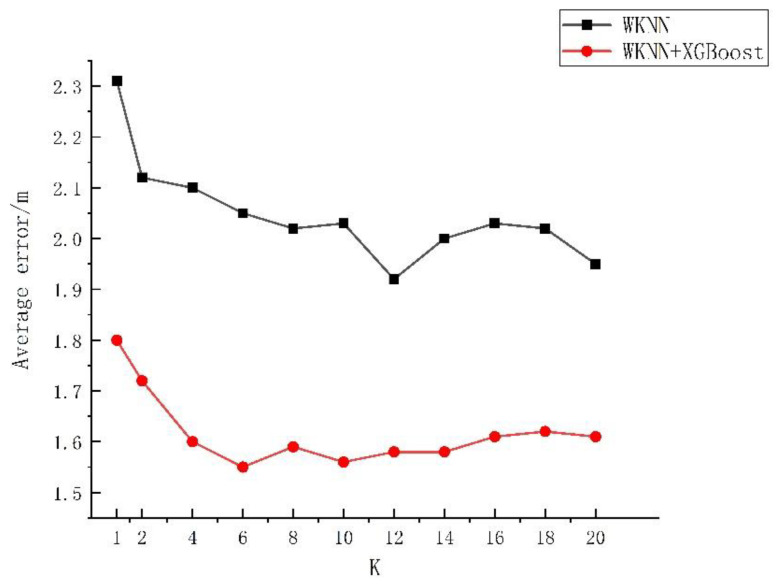
The influence of K value on error.

**Figure 5:**
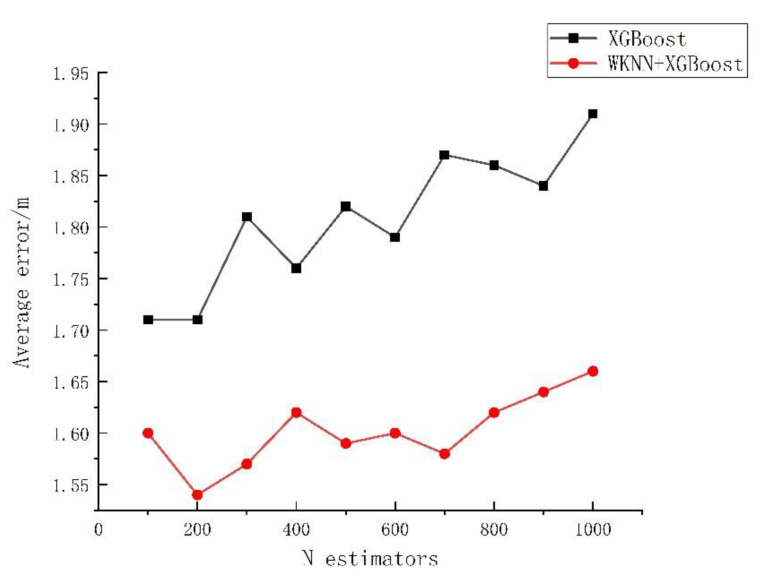
The influence of the number of trees on the error.

**Figure 6:**
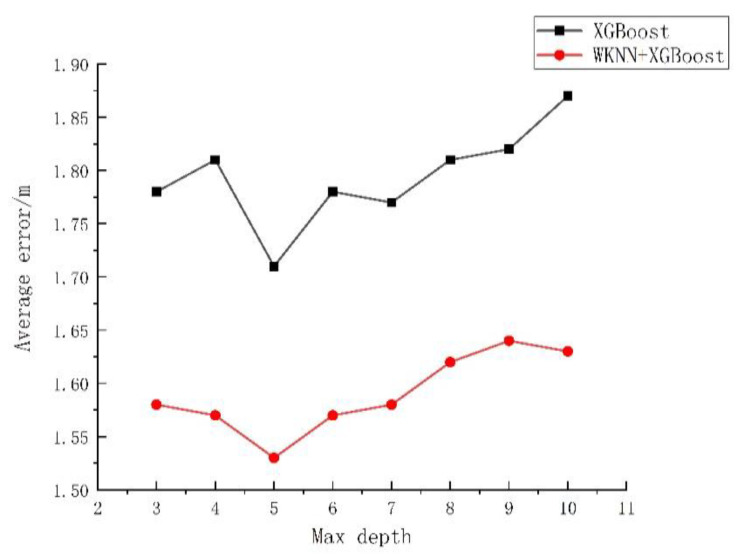
The influence of the depth of the trees on the error.

**Figure 7:**
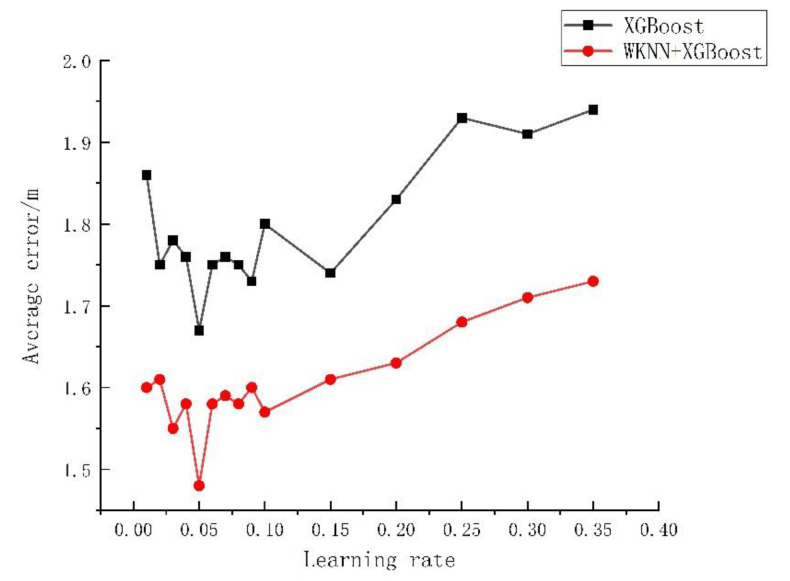
The influence of learning rate on error.

The effect of algorithms can be influenced significantly by algorithm parameters. From the analysis in Section 2.2, it could be known that the final prediction result was influenced greatly by the selected number, depth, and learning rate of trees. Based on the empirical interval of each parameter, in this study the influences of the K value, the number of regression trees, the depth of decision trees, and the learning rate of the traditional WKNN, XGBoost, and WKNN+XGBoost algorithm models on the error were investigated. The experiment was repeated 50 times, and the average value was taken for analysis, with the specific experimental results exhibited in Figure 4, Figure 5, Figure 6 and Figure 7.

As shown in Figure 4, when the K value varied within 1–20, the average positioning error of the WKNN algorithm was 1.92–2.31 m, with a fluctuation of 26.39%. The average positioning error of WKNN and XGBoost algorithms was 1.55–1.8 m, with an overall fluctuation of 11.43%. As shown in Figure 5, when the number of regression trees varied within 100–1000, the average positioning error of the XGBoost algorithm was 1.71–1.91 m, fluctuating by 10.47% as a whole, and that of the WKNN and XGBoost fingerprint positioning algorithms was 1.54–1.66 m, with an overall fluctuation of 8.82%. As shown in Figure 6, within the decision tree depth of 3–10, the average positioning error of the XGBoost algorithm was 1.72–1.88 m, with an overall fluctuation of 8.51%, and that of the WKNN and XGBoost algorithms was 1.53–1.64 m, with an overall fluctuation of 7.23%. As shown in Figure 7, as the learning range changed within 0.01–0.35, the average positioning error of the XGBoost algorithm was 1.68–1.95 m, with an overall fluctuation of 13.85%, while that of the WKNN and XGBoost algorithms ranged from 1.48 to 1.73 m, with an overall fluctuation of 14.37%. From the above experiments, it could be seen that the K value exerted a great influence on the WKNN+XGBoost positioning algorithm, with an influencing degree of 11.43%. Within the empirical value interval, moreover, the change in learning rate reached the highest influencing degree of 14.37%, and the influencing degree of the change in decision tree depth was 7.23% while that of the change in the number of regression trees reached 8.82%. During parameter optimization, therefore, the focus could be on optimizing the K value and learning rate. According to the above experimental results, the initial settings for the basic parameters of the KNN, WKNN, XGBoost, and WKNN+XGBoost algorithms to be compared in this study are shown in Table 2.

In the WKNN algorithm, the weight value ω, which was decided by the distance, should be determined according to the distance. For GA, the empirical value of the population size ps was 20–100, that of the crossover probability pc was 0.4–0.99, and that of the mutation probability pm was 0.0001–0.1. The parameters were adjusted based on the empirical range [20], and the specific parameters finally determined are listed in Table 3.

The initialization data in Table 2 and Table 3 are brought into the algorithm model, and the final experimental parameters are obtained through parameter optimization, as shown in in Table 4.

### 4.4. Positioning Error and Experimental Analysis

**Figure 8:**
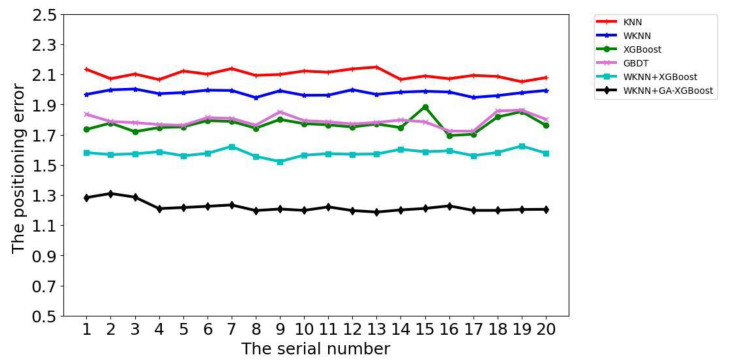
Comparison of positioning error.

**Figure 9:**
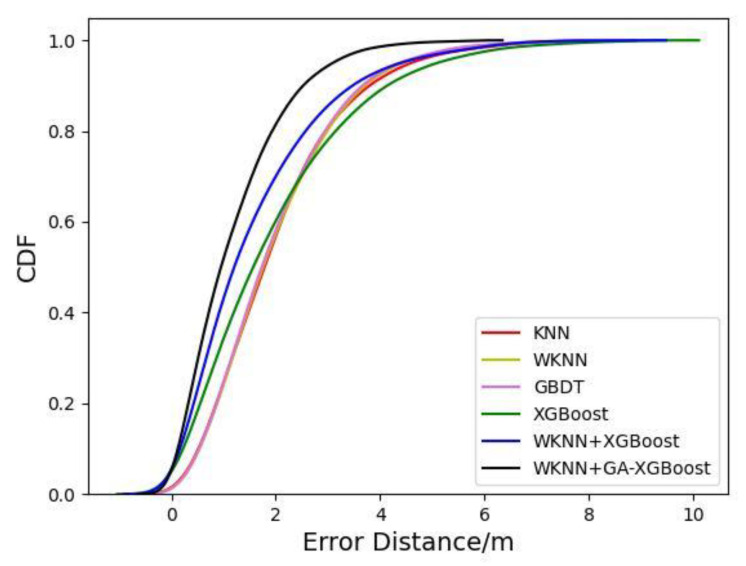
Cumulative probability distribution diagram.

The positioning algorithm based on WKNN and improved XGBoost (WKNN+GA-XGBoost) proposed in this study was compared with the KNN [21], XGBoost [22], GBDT [23], and WKNN positioning algorithms as well as the WKNN and XGBoost fusion algorithm (WKNN+XGBoost without the optimization of XGBoost algorithm parameters), and the comparison diagram of their positioning errors is shown in Figure 8. It could be observed from Figure 8 that under a certain number of training samples, the positioning errors of KNN and XGBoost fingerprint positioning algorithms were 2.05–2.15 m and 1.94–2.01 m, respectively. Additionally, the average positioning errors of XGBoost, WKN+XGBoost, and WKN+GA-XGBoost fingerprint positioning algorithms were 1.70–1.88 m, 1.53–1.62 m, and 1.19–1.31 m, respectively. In the meantime, it could be seen from the CDF diagram in Figure 9 that the WKNN+GA-XGBoost indoor-fingerprint-positioning algorithm could basically predict the actual position of indoor fingerprint points with 100% accuracy at a positioning error of about 4.0 m. At the positioning error of 4.0 m, however, the probability for KNN, WKNN, XGBoost, and WKNN+XGBoost to accurately predict the actual position of indoor fingerprint points was about 90%, 90%, 86%, and 92%, respectively, all of which were lower than that achieved by the proposed WKN+GA-XGBoost indoor-fingerprint-positioning algorithm in this study.

The comparison results of RMSE are listed in Table 5. It could be seen from Table 5 that under a certain number of training samples, the RMSE of KNN, WKNN, XGBoost, GBDT, and WKNN+XGBoost indoor-fingerprint-positioning algorithms was 2.712, 2.673, 2.687, 2.712, and 2.226, respectively, while that of WKNN+GA-XGBoost was 1.665, which was 38.97%, 38.08%, 38.41%, 37.61%, and 25.65% lower than that of KNN, WKNN, XGBoost, GBDT, and WKNN+XGBoost, respectively. Therefore, the WKNN+GA-XGBoost indoor-fingerprint-positioning algorithm proposed in this study exhibited higher positioning performance and practical values.

The comparison results of MAE are shown in Table 6. It could be known from Table 6 that under a certain number of training samples, the MAE of KNN, WKNN, XGBoost, GBDT, and WKNN+XGBoost indoor-fingerprint-positioning algorithms was 2.292, 2.243, 2.102, 2.112, and 1.745, respectively, while that of WKNN+GA-XGBoost was 1.305, which was lower than that of all other algorithms.

## 5. Conclusions

Aiming at the low positioning accuracy and instability of the traditional algorithm KNN, an indoor-fingerprint-positioning algorithm based on WKNN and improved XGBoost was proposed. Firstly, the influences of the K value in the WKNN+XGBoost algorithm and the number of regression trees, the depth of decision trees, and the learning rate in the XGBoost algorithm on indoor-fingerprint-positioning accuracy were discussed. On this basis, then, the parameters in the XGBoost algorithm were dynamically adjusted using GA, and the optimal parameters were found according to the fitness function. Afterward, the nearest neighbor set searched by the WKNN algorithm was introduced into the XGBoost model, and the final predicted coordinates were obtained. Finally, the following conclusions were drawn through experimental verification:

1. In this study, the parameters of the XGBoost algorithm were optimized via GA. It was known through experiments that the XGBoost algorithm was influenced differently by different parameters. In the experiments, the K value and learning rate exerted the greatest influences on the algorithm performance.
2. By fusing WKNN and XGBoost algorithms, an indoor-fingerprint-positioning algorithm based on WKNN+GA-XGBoost was put forward in this study. The experimental results revealed that compared with the other five algorithms, the proposed algorithm reduced the positioning error by 20.26–45.58%, with the RMSE and MAE reduced by over 25%. Moreover, the CDF curve of this algorithm could converge faster.
3. The algorithm proposed in this paper has certain advantages in positioning accuracy and stability. In addition, the fingerprint database was manually acquired offline, which consumed a large quantity of manpower and material resources and led to the failure of real-time updating. Hence, how to simplify the fingerprint database while maintaining a high-accuracy positioning effect remains to be further explored. The experimental results in this article have slightly higher errors. After analysis, the main reason is that the performance of the AP device is weak and the anti-interference ability is poor. We will correct it in future research. Only the positioning of fixed targets in the indoor environment was investigated in this study. The future research focus will be shifted to the positioning of moving targets in indoor environments.

## Figures and Tables

**Table 1:** Composition of the fingerprint database.

ID	AP_1_	AP_2_	AP_3_	AP_4_	AP_5_	x	y
RP_1_ RP_2_ RP_3_ …	−37.2 −34.7 −32.9 …	−57.7 −53.7 −65.3 …	−68.2 −57.5 −71.2 …	−70.4 −57.4 −53.9 …	−55.2 −57.5 −55.2 …	10 20 30 …	10 10 10 …

**Table 2:** Parameter settings of the algorithms.

Algorithm	Parameter	Parameter Specific Numerical Value
WKNN	K	12
KNN	K	12
XGBoost	n_estimators max_depth learning_rate	200 5 0.05
WKNN+ XGBoost	K n_estimators max_depth learning_rate	12 200 5 0.05

**Table 3:** Parameter settings of genetic algorithm.

Algorithm	Parameter	Parameter Specific Numerical Value
GA	ps pc pm	60 0.5 0.01

**Table 4:** Optimal parameters.

Parameter	Numerical Value
K n_estimators max_depth learning_rate	6 210 5 0.05

**Table 5:** RMSE Comparison.

Algorithm	RMSE	Test Set Quantity
KNN WKNN XGBoost GBDT WKNN + XGBoost WKNN + GA- XGBoost	2.712 2.673 2.687 2.712 2.226 1.655	1000 1000 1000 1000 1000 1000

**Table 6:** MAE Comparison.

Algorithm	MAE	Test Set Quantity
KNN WKNN XGBoost GBDT KNN+XGBoost WKNN+GA-XGBoost	2.292 2.243 2.102 2.112 1.745 1.305	1000 1000 1000 1000 1000 1000

## Data Availability

The data presented in this study are available on request from Haizhao Lu.
